# Quantum Chemical Investigation of Polychlorinated Dibenzodioxins, Dibenzofurans and Biphenyls: Relative Stability and Planarity Analysis

**DOI:** 10.3390/molecules25235697

**Published:** 2020-12-03

**Authors:** Sopanant Datta, Taweetham Limpanuparb

**Affiliations:** Science Division, Mahidol University International College, Mahidol University, Salaya, Phutthamonthon, Nakhon Pathom 73170, Thailand; sopanant.dat@student.mahidol.edu

**Keywords:** polychlorinated dibenzodioxins (PCDDs), polychlorinated dibenzofurans (PCDFs), polychlorinated biphenyls (PCBs)

## Abstract

All the possible polychlorinated aromatic compounds in the classes of dibenzodioxins (PCDDs), dibenzofurans (PCDFs), and biphenyls (PCBs) were studied by the quantum chemical methods of HF/6-311++G(d,p), B3LYP/6-311++G(d,p), and MP2/cc-pVTZ. The calculated stabilities and structures of these compounds were compared with the available data on their abundance and toxicity. Prediction models for trends in energy and planarity among these congeners were proposed. The results discussed here can help contribute to the understanding of the role of dioxin-like compounds (DLCs) in the environment.

## 1. Introduction

**Figure 1:**
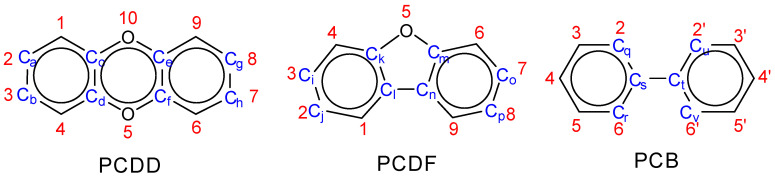
Substitution position numberings (in red) and selected carbon atoms for dihedral angle calculation (in blue) for PCDDs, PCDFs, and PCBs.

The Stockholm Convention on Persistent Organic Pollutants (POPs) [1], initially signed in 2001, includes 12 classes of compounds, three of which can be regarded as dioxin-like compounds (DLCs). (see Figure 1). A subset of DLCs was assigned toxic equivalency factors (TEFs) by the World Health Organization (WHO) [2] to indicate their potential health impacts on humans and mammals. These compounds may accumulate and bioconcentrate through the food chain and, as a result, food products in some jurisdictions such as the European Union are strictly monitored for the presence of these compounds [3]. Quantum chemical methods, semi-empirical calculations, and other related computational techniques have previously been applied to polychlorinated dibenzodioxins (PCDDs), polychlorinated dibenzofurans (PCDFs), and polychlorinated biphenyls (PCBs) [4,5,6,7,8,9,10,11,12,13,14,15,16,17,18,19,20,21,22,23,24,25,26,27,28,29,30,31,32,33,34,35,36]. The goals of these studies were, primarily, to predict their chemical properties and understand their mechanisms of action or degradation in biotic systems. A summary can be found in Supplementary Table S1.

This communication describes the semi-automated codes for generating chemical structures, to be used as inputs for quantum chemical calculations in a standard package and to extract information from outputs for further analysis. Three different representative quantum chemical methods were selected for comparison: Hartree-Fock (HF), Density Functional Theory (DFT), and second-order Møller–Plesset perturbation theory (MP2). The lists of 76 PCDDs, 136 PCDFs, and 210 PCBs (inclusive of three non-chlorinated base structures) along with their systematic numberings [37,38,39] are shown in Supplementary Table S2–S4, respectively. The use of numberings herein will be in accordance with those in the lists. (This is not to be confused with PubChem ID in the Supplementary Materials and elution order number which is instrument-specific.) As shown in Supplementary Table S5, the congeners can be categorized into homologue groups by their empirical formulas or degrees of substitution.

To the best of our knowledge, in comparison to the existing literature this is the most comprehensive theoretical study in terms of the number of compounds in the study and the level of theory employed for the investigation. The computational results are presented in terms of the relative stability and planarity of compounds. These calculated results are compared to the experimental results in the literature in terms of their isomer distribution and toxicity. A better understanding of the nature of these compounds can lead us to a better management of and more sustainable solutions for POPs in the future.

## 2. Results

### 2.1. Relative Stability

The association between the substitution patterns of each compound and its relative stability, with respect to its isomers, was assessed. The substitution pattern parameters include the degree of chlorination (using the number of H atoms, or the number of Cl atoms subtracted from 10 for PCBs and 8 for PCDDs and PCDFs) and the intra-ring and cross-ring interactions between two substituents. Intra-ring interactions are quantified by the number of pairs of Cl and non-hydrogen substituents (Cl, O, and C) at *o-*, *m-* and *p-* positions on each phenyl ring. Cross-ring interactions are defined as the number of pairs of Cl substitution over an O-bridge (CR-O) and a C-C bond (CR-C). The possible parameters for intra-ring and cross-ring interactions are unique for each class of compounds, as shown in Table 1. The total number of *o-*, *m-* and *p-* pairs were also calculated from the tally of substituent pairs mentioned earlier. Overall, as listed in the table, there are a total of 11, 15, and 11 potential parameters for PCDDs, PCDFs, and PCBs, respectively. The following simple linear model was proposed for a relative stability prediction based on HF, B3LYP ∆*G* at 300 K, and MP2 electronic energy *S* = *x*_0_ + 10^2^*x*_1_ + 10^4^*x*_2_ + 10^6^*x*_3_ + 10^8^*x*_4_ + … where *S* is the energy score and *x*_0_, *x*_1_, *x*_2_, … are possible parameters. Coefficients of 10^2*n*^ are used as some variables can take up to two-digit values (0 to 12 for example). The association between energy scores and the calculated energy values was assessed using Spearman’s correlation coefficient (ρ). Inspired by the knapsack problem, parameters that give the highest ρ values were added to the model one by one, until the increase in the ρ value became less than 0.0001.

Table 2 shows the resulting parameters, along with the corresponding ρ values. The results from all methodologies gave ρ > 0.99, and were mostly in agreement with each other. The major predictor for all compounds, in addition to the degree of substitution, are the intra-ring interactions. The predictor with the highest priority is the number of substituent pairs at *o-* positions, regardless of substituent type, followed by substitutions at the *m-* and *p-* positions, respectively. This supports the expectations of steric hindrance from the substituents (bulkiness: Cl > O ≈ C > H). Further, a cross-ring interaction over a C-C bond is an important parameter, especially for PCDFs, whereas a cross-ring interaction over an O-bridge is of less priority for PCDDs. With these results, the stability ranks of these compounds, regardless of methodology, can be sufficiently explained by their substituent pair interactions. For example, the energy scores *S* of PCDDs-13 and 14, when fitted according to the B3LYP results, can be calculated as:

*S*_PCDD-13_ = *x*_0_ + 10^2^*x*_1_ + 10^4^*x*_2_ + 10^6^*x*_3_ + 10^8^*x*_4_ + 10^10^*x*_5_

= 1 + 10^2^(0) + 10^4^(1) + 10^6^(2) + 10^8^(3) + 10^10^(5)

= 50,302,010,001

*S*_PCDD-14_ = *x*_1_ + 10^2^*x*_2_ + 10^4^*x*_3_+ 10^6^*x*_4_+ 10^8^*x*_5_ + 10^10^*x*_6_

= 2 + 10^2^(1) + 10^4^(1) + 10^6^(1) + 10^8^(3) + 10^10^(5)

= 50,301,010,102, respectively.

PCDD-13 ranks higher than PCDD-14 and is predicted to have a higher energy and less stability.

The weights used in this rank modelling are to provide a non-overlapping priority of consideration for each predictor variable. Qualitative interpretation then becomes obvious. Multiple linear regression for the prediction of energy values, following a similar knapsack approach, was also explored and is shown in Supplementary Table S6. In this case, the priorities of the predictors overlap and the already high ρ values can be increased further. Our computational model is able to predict the relative stabilities of all of these classes of compounds with a stronger correlation than the linear models proposed earlier [16,17,18,19], while not increasing the numbers of parameters.

### 2.2. Isomer Distribution

The energetic data in the previous subsection were used to predict the distribution of isomers within each homologue group. For this analysis, energy values were used to evaluate the distribution of isomers using the Boltzmann distribution at 300, 600, and 900 K [40]. The results were compared to available data on the abundance of these compounds in nature [37,40,41,42,43,44,45,46,47,48,49,50,51,52,53,54,55,56]. A brief review of these data in the literature is shown in Supplementary Table S7. Our PCDDs and PCDFs results agree with a limited number of reports on experimental isomer distribution from incineration sources, while no clear trends were observed for PCBs (see Supplementary Table S8. The median ρ values calculated for each homologue group are 0.5833, 0.6555, and −0.1044 for PCDDs, PCDFs, and PCBs, respectively). For all three classes, the distribution of compounds found in nature usually differ due to the different accumulation and decomposition mechanisms in biotic and abiotic sources. For PCBs, it was argued that the distribution of isomers from the source is kinetically controlled rather than thermodynamically controlled [26].

### 2.3. Planarity

Planarity is widely claimed to be an important factor for these compounds to bind with the aryl hydrocarbon receptor (AhR) [57]. Therefore, geometric data were extracted and selected dihedral angles were calculated to represent the coplanarity of two phenyl rings. The designated carbon atoms for dihedral angle calculation are shown in Figure 1 and the list below.



**PCDD**

**PCDF**

**PCB**
C_a_,C_c_,C_d_,C_h_ C_b_,C_d_,C_c_,C_g_ C_a_,C_e_,C_f_,C_h_ C_b_,C_f_,C_e_,C_g_C_a_,C_d_,C_f_,C_g_ C_b_,C_c_,C_e_,C_h_C_i_,C_k_,C_l_,C_p_ C_j_,C_l_,C_k_,C_o_ C_i_,C_m_,C_n_,C_p_ C_i_,C_n_,C_m_,C_o_C_i_,C_l_,C_n_,C_o_ C_j_,C_k_,C_m_,C_p_C_q_,C_s_,C_t_,C_u_ C_q_,C_s_,C_t_,C_v_ C_r_,C_s_,C_t_,C_u_ C_r_,C_s_,C_t_,C_v_


The lists for PCDDs and PCDFs are separated into two groups representing two orthogonal axes of rotation. Absolute values of the acute angle representations of the angles were used for the mean calculation of each group.

Our data show that most PCDDs are planar, and therefore the data are insufficient for further prediction.

For PCDFs, with a limited number of compounds exhibiting non-planarity, there appears to be a moderate trend (*R* values ranging from 0.61 to 0.65) between the substitution pattern and coplanarity. The significant parameters (*p* < 0.05) involved are the presence or absence of a substituent pair at positions 1 and 9 and at positions 2 and 8. The reasons for this finding may be unclear due to a limited number of data points (*n* = 13) for analysis and moderate correlation. The prediction models were derived from multiple linear regression and are shown in Table S9.

For PCBs, a dihedral angle prediction, based on chlorine substitution position, was also assessed using multiple linear regression. In this model, the dihedral angle *A* of a compound is predicted by the equation:

*A* = *C*_0_ + *C*_1_(*x*_2_ + *x*_2′_ + *x*_6_ + *x*_6′_) + *C*_2_(*x*_2_ + *x*_6_)(*x*_2′_ + *x*_6′_) + *C*_3_(*x*_3_ + *x*_3′_ + *x*_5_ + *x*_5′_) + *C*_4_(*x*_4_ + *x*_4′_)

where; *C*_1_, *C*_2_, *C*_3_, and *C*_4_ are the coefficients to be determined; and *x*_*i*_ is 1 if position *i* is occupied by a Cl atom, and 0 if otherwise.

Table 3 shows the coefficients of significant parameters (*p* < 0.05) and the corresponding correlation coefficients for the predictions. The results from all methodologies show that the most important parameter is Cl substitution at any one of the four positions ortho to the C-C bond connecting two rings, as mentioned in earlier findings [57]. This is then followed by the same kind of substitution on both rings (product of *x*_2_ + *x*_6_ and *x*_2′_ + *x*_6′_). Meta substitutions according to all methods and para substitutions, according to MP2 results, are less important parameters, as *C*_3_ and *C*_4_ are relatively small.

### 2.4. Toxicity

When comparing our geometric data to the toxic equivalency factor (TEF) values of these compounds [2], as shown in Supplementary Table S10, the following points are observed.

- The twelve known dioxin-like PCBs with TEF values are among the ones with relatively small dihedral angles or are closer to being planar.
- According to the B3LYP and MP2 results, there are three non-coplanar PCDDs (PCDDs-46, 65, and 71) from both methods, and two additional (PCDDs-42 and 45) from B3LYP only. All of these are not substituted at all four lateral positions (2,3,7,8), which are the substitution positions of PCDDs known to be toxic [2]. One of these, PCDD-46, is substituted at positions exactly opposite to that of 2,3,7,8-tetrachlorodibenzodioxin (TCDD), the most toxic dioxin-like compound [2].

## 3. Discussion, Conclusions and Future Work

Overall, this study provides high-quality quantum chemical calculations for the energetic and geometric properties of PCDDs, PCDFs, and PCBs. The comprehensive data provided for all of these three related classes of compounds can be used for further investigations. From our computational results, we have proposed prediction models for the important characteristics of these compounds. The relative stability prediction extends beyond earlier prediction models [16,17,18,19] with a high accuracy and with the allowance of qualitative interpretations. Prediction models have also been proposed for planarity, an important structural parameter for the toxic activities of these compounds. Furthermore, we have made associations between theoretical calculations (relative stability and planarity) and experimental data in the literature (abundance and toxicity).

Our work can be used for further analyses on the persistence and activities of the toxic compounds in nature. In terms of methodology, semi-automated codes in the Supplementary Materials can be extended further for the construction and analysis of other related compounds. All three methods, HF, B3LYP, and MP2, yielded similar trends for the classes of compounds in our study. Our results suggest that a computationally expensive treatment for correlation energy may not be needed for this purpose and a low-level method such as HF, in most cases, can sufficiently provide insightful findings on the molecular properties of the studied classes of compounds. The further investigation of different variations of DFT methods may also be useful. For example, range-separated hybrid functionals such as ωB97XD can be used for comparison with the results presented here.

## 4. Materials and Methods

We extended our exhaustive combinatorial investigation approach [58,59,60] to all members of the three classes of compounds. The Becke three-parameter hybrid functional combined with the Lee-Yang-Parr correlation functional (B3LYP), the most popular variation of DFT, was selected for this work. In total, three methods, HF/6-311++G(d,p), B3LYP/6-311++G(d,p), and MP2/cc-pVTZ [61], were used on Q-Chem 5.1 (developer version) [62] for geometry optimization. The HF- and B3LYP-optimised geometries were confirmed to be minima by verifying the absence of imaginary frequencies. All the raw output files and incomplete attempts made with MP2/6-311++G(d,p) are provided in the Supplementary Materials. The process of the structure enumeration and analysis of molecular properties, particularly relative energy and planarity, were completed by semi-automated scripts written in Mathematica for this project. The Mathematica notebook can be found in the Supplementary Materials. These Mathematica codes can be used for the construction and analysis of other compounds and for teaching purposes in classes relating to molecular modelling and cheminformatics. Our computational results were compared to the relative abundance of the compounds in nature and their estimated toxicity in humans and animals. The analyses presented are derived from the energetic and geometric data extracted from the computational results.

For energetic information, the electronic energy of each compound was obtained from the results of all methodologies and, additionally, the enthalpy, entropy, and Gibbs energy values were obtained from the frequency jobs of HF and B3LYP. Due to the free rotation about C-C bonds connecting the two phenyl rings in PCBs, 78 potential atropisomers [38] were explored and only the structures with the lower energy values were used for further calculations.

## Figures and Tables

**Table 1:** Possible numbers of substituent pairs for each compound.

Substitution Positions	Substituent Pair	PCDD	PCDF	PCB
Intra-Ring Interactions				
*o-*	Cl-Cl	0–6 ^1^	0–6	0–8
	Cl-O	0–4	0–2	N/A
	Cl-C	N/A	0–2	0–4
	Total	0–10	0–10	0–12
*m-*	Cl-Cl	0–4	0–4	0–8
	Cl-O	0–8	0–4	N/A
	Cl-C	N/A	0–4	0–4
	Total	0–12	0–12	0–12
*p-*	Cl-Cl	0–2	0–2	0–4
	Cl-O	0–4	0–2	N/A
	Cl-C	N/A	0–2	0–2
	Total	0–6	0–6	0–6
**Cross-ring interactions**				
Over O-bridge	Cl-Cl	0–2 ^2^	0–1 ^3^	N/A
Over C-C bond		N/A	0–1 ^3^	0–4 ^4^

^1^ Example: A fully substituted PCDD (PCDD-75) has six *o*-Cl-Cl pairs at positions (1,2), (2,3), (3,4), (6,7), (7,8), and (8,9). Possible pairs are substitutions at positions: ^2^ (1,9) and (4,6); ^3^ (4,6) over O-bridge and (1,9) over C-C bond; ^4^ (2,2′), (2,6′), (2′,6), and (6,6′) due to the free rotation about the C-C bond connecting two rings. N/A indicates a case whereby such a substituent pair is not possible.

**Table 2:** Parameters (in order of *x*_0_, *x*_1_, *x*_2_, …) for relative stability prediction and the corresponding correlation coefficients (ρ).

PCDD	HF		B3LYP		MP2	
Parameters	CR-	O	CR-	O	CR-	O
	*p-*	Cl-Cl	*p-*	Cl-Cl	*p-*	Cl-Cl
	*m-*	Cl-Cl	*m-*	Cl-Cl	*m-*	Cl-Cl
	*o-*	Cl-Cl	*o-*	Cl-Cl	*o-*	Cl-Cl
	*o-*	total	*o-*	total	*o-*	total
	total H		total H		total H	
ρ	0.9998		0.9997		0.9996	
**PCDF**	**HF**		**B3LYP**		**MP2**	
Parameters	*p*-	Cl-O	*m*-	Cl-Cl	*m*-	Cl-Cl
	*m*-	Cl-Cl	*p*-	Cl-O	*p*-	Cl-Cl
	*o*-	Cl-O	*o*-	Cl-O	*p*-	Cl-O
	*o*-	Cl-Cl	*o*-	Cl-Cl	*o*-	Cl-O
	CR-	C	CR-	C	*o*-	Cl-Cl
	total H		*o*-	total	CR-	C
			total H		total H	
ρ	0.9997		0.9962		0.9978	
**PCB**	**HF**		**B3LYP**		**MP2**	
Parameters	*p-*	total	*m-*	total	*p-*	total
	*m-*	total	*o-*	Cl-Cl	*m-*	total
	*o-*	Cl-Cl	*o-*	total	*o-*	Cl-Cl
	*o-*	total	total H		total H	
	total H					
ρ	0.9993		0.9990		0.9978	

**Table 3:** Constants, coefficients, and Pearson’s (*R*) and Spearman’s (ρ) correlation coefficients for the PCB planarity prediction model.

	Coefficients					*R*	ρ
	*C*_0_	*C*_1_	*C*_2_	*C*_3_	*C*_4_		
HF	50.858	20.325	−10.751	0.724	N/A	0.9354	0.8735
B3LYP	37.926	24.996	−11.419	0.529	N/A	0.9552	0.9599
MP2	33.308	24.202	−9.499	0.668	−0.529	0.9800	0.9560

N/A indicates a case whereby the coefficient is not required and can be regarded as zero.

## Supplementary Materials

The following are available online, Table S1: Summary of computational data and analyses on PCDDs, PCDFs, and PCBs in the literature; Table S2: List of all PCDDs by substitution positions and numbering, proposed by Ballschmiter et al., revised by Dorofeeva et al.; Table S3: List of all PCDFs by substitution positions and numbering, proposed by Ballschmiter et al.; Table S4: List of all PCBs by substitution positions and numbering, first proposed by Ballschmiter et al., later revised by IUPAC; Table S5: List of congener groups of PCDDs, PCDFs and PCBs by degree of substitution; Table S6: Values for constant *C*_0_ and coefficients *C*_1_, *C*_2_, *C*_3_,…, parameters *x*_1_, *x*_2_, *x*_3_,… and correlation coefficients for the energy prediction model *E* = *C*_0_ + *C*_1_*x*_1_ + *C*_2_*x*_2_ + *C*_3_*x*_3_ + …, where *E* is the predicted HF, B3LYP ∆*G* at 300 K in Hartree and MP2 electronic energy in Hartree; Table S7: Summary of experimental data on abundance of PCDDs, PCDFs and PCBs in the literature; Table S8: Rank correlation coefficients (ρ values) for association between predicted distribution (using Boltzmann distribution) of PCDDs and PCDFs based on relative stability and observed distribution in fly ash samples from municipal waste incinerators (MWI); Table S9: Values for constant *C*_0_, coefficients *C*_1_, *C*_2_, and correlation coefficients for the PCDFs coplanarity prediction model; Table S10: List of toxic equivalency factor (TEF) values of 7 PCDDs, 10 PCDFs and 12 PCBs adapted from Van den Berg et al.

## References

[1] United Nations (2006). Treaties and International Agreements Registered or Filed and Recorded with the Secretariat of the United Nations.

[2] Van den Berg M., Birnbaum L.S., Denison M., De Vito M., Farland W., Feeley M., Fiedler H., Hakansson H., Hanberg A., Haws L. (2006). The 2005 World Health Organization reevaluation of human and mammalian toxic equivalency factors for dioxins and dioxin-like compounds. Toxicol. Sci..

[3] European Commission (2006). Commission Regulation (EC) No 1881/2006 of 19 December 2006 setting maximum levels for certain contaminants in foodstuffs. Off. J. Eur. Union.

[4] Arulmozhiraja S., Fujii T., Tokiwa H. (2000). Electron affinity for the most toxic 2,3,7,8-tetrachlorodibenzo-*p*-dioxin (TCDD):  A density functional theory study. J. Phys. Chem. A.

[5] Dorofeeva O.V., Moiseeva N.F., Yungman V.S., Novikov V.P. (2001). Ideal gas thermodynamic properties of biphenyl. Thermochim. Acta.

[6] Iino F., Tsuchiya K., Imagawa T., Gullett B.K. (2001). An isomer prediction model for PCNs, PCDD/Fs, and PCBs from municipal waste incinerators. Environ. Sci. Technol..

[7] Arulmozhiraja S., Fujii T., Morita M. (2002). Density functional theory studies on radical ions of selected polychlorinated biphenyls. J. Phys. Chem. A.

[8] Chana A., Concejero M.A., de Frutos M., González M.J., Herradón B. (2002). Computational studies on biphenyl derivatives. Analysis of the conformational mobility, molecular electrostatic potential, and dipole moment of chlorinated biphenyl:  Searching for the rationalization of the selective toxicity of polychlorinated biphenyls (PCBs). Chem. Res. Toxicol..

[9] León L.A., Notario R., Quijano J., Sánchez C. (2002). Structures and enthalpies of formation in the gas phase of the most toxic polychlorinated dibenzo-*p*-dioxins. A DFT study. J. Phys. Chem. A.

[10] Dorofeeva O.V., Yungman V.S. (2003). Enthalpies of formation of dibenzo-*p*-dioxin and polychlorinated dibenzo-*p*-dioxins calculated by density functional theory. J. Phys. Chem. A.

[11] Lee J.-E., Choi W. (2003). DFT calculation on the electron affinity of polychlorinated dibenzo-*p*-dioxins. Bull. Korean Chem. Soc..

[12] Lee J.E., Choi W., Mhin B.J. (2003). DFT calculation on the thermodynamic properties of polychlorinated dibenzo-*p*-dioxins:  intramolecular Cl−Cl repulsion effects and their thermochemical implications. J. Phys. Chem. A.

[13] Arulmozhiraja S., Morita M. (2004). Electron affinities and reductive dechlorination of toxic polychlorinated dibenzofurans: A density functional theory study. J. Phys. Chem. A.

[14] Dorofeeva O.V., Moiseeva N.F., Yungman V.S. (2004). Thermodynamic properties of polychlorinated biphenyls in the gas phase. J. Phys. Chem. A.

[15] Ryu J.-Y., Mulholland J.A., Dunn J.E., Iino F., Gullett B.K. (2004). Potential role of chlorination pathways in PCDD/F formation in a municipal waste incinerator. Environ. Sci. Technol..

[16] Wang Z.-Y., Zhai Z.-C., Wang L.-S., Chen J.-L., Kikuchi O., Watanabe T. (2004). Prediction of gas phase thermodynamic function of polychlorinated dibenzo-*p*-dioxins using DFT. J. Mol. Struc. Theochem.

[17] Wang Z.-Y., Zhai Z.-C., Wang L.-S. (2005). Prediction of gas phase thermodynamic properties of polychlorinated dibenzo-furans by DFT. J. Mol. Struc. Theochem.

[18] Wang Z.Y., Han X.Y., Zhai Z.C., Wang L.S. (2005). Study on the thermodynamic property and relative stability of a series of polychlorinated biphenyls by density functional theory. Acta Chim. Sinica.

[19] Zhai Z.-C., Wang Z.-Y., Chen X.-H., Wang L.-S. (2005). DFT calculation on 204 polychlorinated biphenyls: Their thermodynamic function and implication of Cl substitute position. J. Mol. Struc. Theochem.

[20] Dorofeeva O.V., Vishnevskiy Y.V., Moiseeva N.F. (2006). Assessment of Gaussian-3X theory for chlorinated organic molecules. Enthalpies of formation of chlorobenzenes and predictions for polychlorinated aromatic compounds. Struct. Chem..

[21] Thompson D., Ewan B.C.R. (2007). A group additivity algorithm for polychlorinated dibenzofurans derived from selected DFT analyses. J. Phys. Chem. A.

[22] Wang L., Heard D.E., Pilling M.J., Seakins P. (2008). A Gaussian-3X prediction on the enthalpies of formation of chlorinated phenols and dibenzo-*p*-dioxins. J. Phys. Chem. A.

[23] Jansson S., Antti H., Marklund S., Tysklind M. (2009). Multivariate relationships between molecular descriptors and isomer distribution patterns of PCDD/Fs formed during MSW combustion. Environ. Sci. Technol..

[24] Wang L., He Y.-L. (2009). The enthalpies of formation for polychlorinated dibenzofurans with use of G3XMP2 model chemistry and density functional theory. J. Phys. Chem. A.

[25] Ewan B.C.R., Thompson D. (2013). Predicted specific heat behaviour of the polychlorinated dibenzofuran family from DFT analysis. Thermochim. Acta.

[26] Jansson S., Grabic R. (2014). Multivariate relationships between molecular descriptors and isomer distribution patterns of PCBs formed during household waste incineration. Environ. Sci. Pollut. Res..

[27] Zhou Q., Su X., Yong Y., Ju W., Fu Z., Li X. (2018). Adsorption of 2, 3, 7, 8-tetrachlorodibenzao-*p*-dioxin (TCDD) on graphane decorated with Ni and Cu: A DFT study. Vacuum.

[28] Abronin I.A., Volkova L.V. (2019). On the relationship between the energy characteristics of the isodesmic reactions of polychlorinated dioxins and their toxicity. Russ. Chem. Bull..

[29] Bai N., Wang W., Zhao Y., Feng W., Li P. (2019). Theoretical insights into the reaction mechanism between 2,3,7,8-tetrachlorodibenzofuran and hydrogen peroxide: A DFT study. ACS Omega.

[30] Hou S., Altarawneh M., Kennedy E.M., Mackie J.C., Weber R., Dlugogorski B.Z. (2019). Formation of polychlorinated dibenzo-*p*-dioxins and dibenzofurans (PCDD/F) from oxidation of 4,4′-dichlorobiphenyl (4,4′-DCB). Proc. Combust. Inst..

[31] Behjatmanesh-Ardakani R., Heydari A. (2020). Molecular and dissociative adsorption of tetrachlorodibenzodioxin on M-doped graphenes (M = B, Al, N, P): Pure DFT and DFT + VdW calculations. J. Mol. Model..

[32] Diao J., Li Y., Shi S., Sun Y., Sun Y. (2010). QSAR models for predicting toxicity of polychlorinated dibenzo-*p*-dioxins and dibenzofurans using quantum chemical descriptors. Bull. Environ. Contam. Toxicol..

[33] Larsson M., Kumar Mishra B., Tysklind M., Linusson A., Andersson P.L. (2013). On the use of electronic descriptors for QSAR modelling of PCDDs, PCDFs and dioxin-like PCBs. SAR QSAR Environ. Res..

[34] Hirano M., Hwang J.-H., Park H.-J., Bak S.-M., Iwata H., Kim E.-Y. (2015). In silico analysis of the interaction of avian aryl hydrocarbon receptors and dioxins to decipher isoform-, ligand-, and species-specific activations. Environ. Sci. Technol..

[35] Larsson M., Fraccalvieri D., Andersson C.D., Bonati L., Linusson A., Andersson P.L. (2018). Identification of potential aryl hydrocarbon receptor ligands by virtual screening of industrial chemicals. Environ. Sci. Pollut. Res..

[36] Zeinali N., Oluwoye I., Altarawneh M., Dlugogorski B.Z. (2019). Destruction of dioxin and furan pollutants via electrophilic attack of singlet oxygen. Ecotoxicol. Environ. Saf..

[37] Ballschmiter K., Buchert H., Niemczyk R., Munder A., Swerev M. (1986). Automobile exhausts versus municipal-waste incineration as sources of the polychloro-dibenzodioxins (PCDD) and -furans (PCDF) found in the environment. Chemosphere.

[38] Agudo A., Aronson K.J., Bonefeld-Jorgensen E.C., Cocco P., Cogliano V., Cravedi J.-P., Esch H., Fiedler H., Glauert H.P., Guo Y.-L.L. (2016). Polychlorinated Biphenyls and Polybrominated Biphenyls.

[39] Ballschmiter K., Zell M. (1980). Analysis of polychlorinated biphenyls (PCB) by glass capillary gas chromatography. Fresenius Z. Anal. Chem..

[40] Dickson L.C., Lenoir D., Hutzinger O. (1992). Quantitative comparison of de novo and precursor formation of polychlorinated dibenzo-*p*-dioxins under simulated municipal solid waste incinerator postcombustion conditions. Environ. Sci. Technol..

[41] Tiernan T.O., Taylor M.L., Garrett J.H., VanNess G.F., Solch J.G., Deis D.A., Wagel D.J. (1983). Chlorodibenzodioxins, chlorodibenzofurans and related compounds in the effluents from combustion processes. Chemosphere.

[42] Schwartz T.R., Campbell R.D., Stalling D.L., Little R.L., Petty J.D., Hogan J.W., Kaiser E.M. (1984). Laboratory data base for isomer-specific determination of polychlorinated biphenyls. Anal. Chem..

[43] Ballschmiter K., Swerev M. (1987). Reaction pathways for the formation of polychlorodibenzodioxins (PCDD) and -furans (PCDF) in combustion processes I. Fresenius Z. Anal. Chem..

[44] Yasuhara A., Ito H., Morita M. (1987). Isomer-Specific Determination of polychlorinated dibenzo-*p*-dioxins and dibenzofurans in incinerator-related environmental samples. Environ. Sci. Technol..

[45] Faroon O.M., Samuel Keith L., Smith-Simon C., De Rosa C.T. (2003). Polychlorinated Biphenyls: Human Health Aspects.

[46] Hanari N., Horii Y., Okazawa T., Falandysz J., Bochentin I., Orlikowska A., Puzyn T., Wyrzykowska B., Yamashita N. (2004). Dioxin-like compounds in pine needles around Tokyo Bay, Japan in 1999. J. Environ. Monit..

[47] Kim K.S., Hirai Y., Kato M., Urano K., Masunaga S. (2004). Detailed PCB congener patterns in incinerator flue gas and commercial PCB formulations (Kanechlor). Chemosphere.

[48] Haws L.C., Su S.H., Harris M., DeVito M.J., Walker N.J., Farland W.H., Finley B., Birnbaum L.S. (2006). Development of a refined database of mammalian relative potency estimates for dioxin-like compounds. Toxicol. Sci..

[49] Ishikawa Y., Noma Y., Yamamoto T., Mori Y., Sakai S.-i. (2007). PCB decomposition and formation in thermal treatment plant equipment. Chemosphere.

[50] Srogi K. (2008). Levels and congener distributions of PCDDs, PCDFs and dioxin-like PCBs in environmental and human samples: A review. Environ. Chem. Lett..

[51] Sundqvist K.L., Tysklind M., Geladi P., Hopke P.K., Wiberg K. (2010). PCDD/F source apportionment in the Baltic sea using positive matrix factorization. Environ. Sci. Technol..

[52] Jansson S., Lundin L., Grabic R. (2011). Characterisation and fingerprinting of PCBs in flue gas and ash from waste incineration and in technical mixtures. Chemosphere.

[53] Barahona-Urbina C., Nunez-Gonzalez S., Gomez-Jeria J.S. (2012). Model-based quantum-chemical study of the uptake of some polychlorinated pollutant compounds by zucchini subspecies. J. Chil. Chem. Soc..

[54] Hinwood A.L., Callan A.C., Heyworth J., Rogic D., de Araujo J., Crough R., Mamahit G., Piro N., Yates A., Stevenson G. (2014). Polychlorinated biphenyl (PCB) and dioxin concentrations in residential dust of pregnant women. Environ. Sci. Process. Impacts.

[55] Thompson D., Ooi T.C., Anderson D.R., Fisher R., Ewan B.C.R. (2016). The polychlorinated dibenzofuran fingerprint of iron ore sinter plant: Its persistence with suppressant and alternative fuel addition. Chemosphere.

[56] Huang R., Wang P., Zhang J., Chen S., Zhu P., Huo W., Jiang Y., Chen Z., Peng J. (2019). The human body burden of polychlorinated dibenzo-*p*-dioxins/furans (PCDD/Fs) and dioxin-like polychlorinated biphenyls (DL-PCBs) in residents’ human milk from Guangdong Province, China. Toxicol. Res..

[57] The Eightieth Meeting of the Joint FAO/WHO Expert Committee on Food Additives (JECFA) (2016). Safety Evaluation of Certain Food Additives and Contaminants, Supplement 1: Non-Dioxin-Like Polychlorinated Biphenyls.

[58] Datta S., Limpanuparb T. (2020). Geometric and energetic data from quantum chemical calculations of halobenzenes and xylenes. Data Brief.

[59] Limpanuparb T., Datta S., Chinsukserm K., Teeraniramitr P. (2020). In silico geometric and energetic data of all possible simple rotamers made of non-metal elements. Data Brief.

[60] Chinsukserm K., Lorpaiboon W., Teeraniramitr P., Limpanuparb T. (2019). Geometric and energetic data from ab initio calculations of haloethene, haloimine, halomethylenephosphine, haloiminophosphine, halodiazene, halodiphosphene and halocyclopropane. Data Brief.

[61] Riley K.E., Hobza P. (2007). Assessment of the MP2 method, along with several basis sets, for the computation of interaction energies of biologically relevant hydrogen bonded and dispersion bound complexes. J. Phys. Chem. A.

[62] Shao Y., Gan Z., Epifanovsky E., Gilbert A.T., Wormit M., Kussmann J., Lange A.W., Behn A., Deng J., Feng X. (2015). Advances in molecular quantum chemistry contained in the Q-Chem 4 program package. Mol. Phys..

