# Supplementary material for: Quantum Chemical Investigation of Polychlorinated Dibenzodioxins, Dibenzofurans and Biphenyls: Relative Stability and Planarity Analysis
Source: Molecules. 2020 Dec 3;25(23):5697. doi: 10.3390/molecules25235697 (PMC7730433; doi:10.3390/molecules25235697)
Supplement: Supplementary file 1 [file molecules-25-05697-s001.zip › SI_Description.pdf]

Quantum Chemical Investigation of Polychlorinated Dibenzodioxins, Dibenzofurans and Biphenyls: Relative Stability and Planarity Analysis

Sopanant Datta and Taweetham Limpanuparb *
Science Division, Mahidol University International College, Mahidol University, Salaya, Phutthamonthon, Nakhon Pathom 73170, Thailand; sopanant.dat@student.mahidol.edu
* Correspondence: taweetham.lim@mahidol.edu

DOI: 10.3390/molecules25235697

- Supplementary Tables.pdf contains supplementary tables referred to by the manuscript.
- 2D-NumberingTables.xlsx contains tables showing the association between compound numbering and chlorination positions on each phenyl ring, with separated sheets for PCDDs, PCDFs and PCBs.
- ExampleGraphs.xlsx contains examples of tables and graphs showing the association between predicted and calculated values of planarity (for PCBs, based on B3LYP results) and energy (for PCDDs, based on MP2 results).
- EnergyPrediction.nb (Mathematica notebook) contains codes used for energy prediction models for all classes of compounds.
- Script.txt contains scripts used for input file generation and data extraction from output files.

Each of the 3 subfolders for PCDD, PCDF, PCB contains 4 subfolders for data from HF, B3LYP, MP2 methodologies, and raw output files of incomplete attempts from MP2/6-311G++(d,p), and the following:

- cidtable.csv (separated into 0cidtable.csv and 1cidtable.csv for PCBs) showing PubChem CID for all compounds and associated systematic numberings and structures
- mathematica notebook (.nb) with codes used for calculations and analyses
- template files used to generate input files for calculation by each method
- 3 files of Energy_SubPattern_(method: HF/B3LYP/MP2).xlsx, each containing a list of all compounds, their energy values from the associated method and calculated values for each substitution pattern parameter (Table 1 in manuscript) exported from Mathematica
- 3 files of IsomerDist_(300/600/900)K.csv containing a list of all compounds, their relative energy and predicted relative abundance (within their homologue groups) exported from Mathematica. Column headings are shown below:

Compound No.	Degree of substitution	Structure	HF		B3LYP		MP2	
			E_{rel}	P	E_{rel}	P	E_{rel}	P

  - $E_{rel} = E - E_{min}$, where E_{rel} is the relative energy in kcal mol^{-1}, E is the raw energy value and E_{min} is the minimum energy value within the homologue group.
  - P is the relative abundance calculated using E_{rel} and Boltzmann distribution.
- for PCB subfolder only, PCB_isomerdist_correlation.xlsx containing a table of Spearman's correlation coefficients for association between predicted (based on calculated relative energy – HF, B3LYP ΔG at 600K and MP2 electronic energy) and observed distribution (combustion experiments) of PCBs.

Each subfolder of HF, B3LYP and MP2 contains:

- raw output files (.out)
- in not_used subfolder: not used .out files (78 potential atropisomers for PCB)
- energetic data for all compounds:
  - 1_final_energy.csv showing electronic energy in Hartree
  - 3_zero_point.csv showing zero-point vibrational energy in kcal mol^{-1} (HF and B3LYP only)
  - 4_enthalpy.csv showing enthalpy values in kcal mol^{-1} (HF and B3LYP only)
  - 5_entropy.csv showing entropy values in cal mol^{-1} K^{-1}
- geometric data for all compounds:
  - 6_dipole_moment.csv showing dipole moments of optimized structures for all compounds
  - in XYZ subfolder: geometry in Z-matrix and Cartesian coordinate format (.xyz)
  - in XYZ subfolder: 9_geotable.csv showing dihedral angles for all compounds (table contains compound no., substituents in order, dihedral angles as listed in section 2.2 in the manuscript)
