# Supplementary material for: Quantum Chemical Investigation of Polychlorinated Dibenzodioxins, Dibenzofurans and Biphenyls: Relative Stability and Planarity Analysis
Source: Molecules. 2020 Dec 3;25(23):5697. doi: 10.3390/molecules25235697 (PMC7730433; doi:10.3390/molecules25235697)
Supplement: Supplementary file 1 [file molecules-25-05697-s001.zip › Supplementary Tables.pdf]

Quantum Chemical Investigation of Polychlorinated Dibenzodioxins, Dibenzofurans and Biphenyls: Relative Stability and Planarity Analysis

Sopanant Datta and Taweetham Limpanuparb *
Science Division, Mahidol University International College, Mahidol University, Salaya, Phutthamonthon, Nakhon Pathom 73170, Thailand; sopanant.dat@student.mahidol.edu
* Correspondence: taweetham.lim@mahidol.edu

DOI: 10.3390/molecules25235697 **(For references, please see the paper.)**

Supplementary Table S1 Summary of computational data and analyses on PCDDs, PCDFs and PCBs in the literature

Property		PCDD	PCDF	PCB
Energetic/Thermodynamic data	For all compounds	Results based on<br>B3LYP/6-31G(d,p)[9, 11],<br>B3LYP/6-31G(2df,p)[21],<br>B3LYP/6-311G(d,p)[15],<br>B3LYP/6-311G++(3df,3pd)[21],<br>G3XMP2//B3LYP/6-31G(2df,p)[21]	Results based on<br>B3LYP/6-31G(2df,p)[23],<br>B3LYP/6-311G(d,p)[16],<br>B3LYP/6-311+G(3df,p)//B3LYP/6-31G(d)[24],<br>B3LYP/6-311++G(3df,3pd)[23],<br>MP2/G3MP3Large, G3MP2, G3XMP2[23]	Results based on<br>B3LYP/6-31G(d)[17],<br>B3LYP/6-31G(d,p)[7]<br>(possible atropisomers not treated by QM methods)
	For selected compounds	Results based on<br>B3LYP/6-311G(d,p)//B3LYP/6-31G(d,p)[11],<br>B3LYP/cc-pVTZ//B3LYP/6-31G(d,p)[11],<br>B3LYP/6-311+G(3df,2p)//B3LYP/6-31G(d)[8]<br>B3LYP/6-311+G(3df,2p)//B3LYP/6-31G(d,p)[19],<br>B3LYP, B3P86, B3W91 on aug'-cc-pVDZ basis[3]	Results based on<br>B3LYP/6-31G(d),<br>B3LYP/6-311G(d,p),<br>B3LYP/6-311+G(d,p)<br>B3LYP/6-311+G(2d,2p)[12],<br>B3LYP/6-311+G(3df,p)//B3LYP/6-31G(d)[20]<br>(with additional prediction for others)	Results based on<br>B3LYP/6-31G(d)[18],<br>B3LYP/6-311G(d,p)[13],<br>B3LYP/cc-pVTZ (for biphenyl only)[4]
Structural data	For all compounds			Results based on<br>B3LYP/6-31G(d)[7]
	For selected compounds	Results based on<br>B3LYP/6-31G(d)[8],<br>B3W91/aug'-cc-pVDZ[3]	Results based on<br>B3LYP/6-311+G(2d,2p)[12]	Results based on<br>B3LYP/6-311+G(2d,2p)[6]
Others	Electron affinities	Results based on<br>B3LYP/6-31G(d,p), B3LYP/aug-cc-pVDZ,<br>B3LYP/aug-cc-pVTZ//B3LYP/6-31G(d,p)[10],<br>HF, B3LYP, B3P86, B3PW91 on aug'-cc-pVDZ basis[3]	Results based on<br>B3LYP/6-31G(d),<br>B3LYP/6-311G(d,p),<br>B3LYP/6-311+G(d,p),<br>B3LYP/6-311+G(2d,2p)[12]	Results based on<br>B3LYP/6-311G(d,p),<br>B3LYP/6-311+G-(2d,2p)[6]
	Orbitals/ potentials	HOMO and LUMO energies, ionization potential, and natural bond orbital analysis based on B3LYP/6-31G(d)[8]		Electrostatic potential based on B3LYP/6-31G(d) (possible atropisomers not treated by QM methods)[7]
	Reaction mechanisms	- Formation of PCDD/Fs from PCB oxidation based on M062X/6-311+G(d,p)[29]		
		- Destruction by singlet oxygen based on B3LYP/6-311+g(d,p)[35]		
		- Dissociation based on DFT+VdW/SVP[30]<br>- Detection by metal-doped graphene based on PBE/DNP[26]	- Reaction with hydrogen peroxide based on B3LYP/6-311++G(d,p)[28]	
	Analysis of MWI isomer distribution patterns	- Do not follow chlorination models.[5, 14]<br>- *Meta* substituent pairs are influential, suggests chlorophenols as precursors.[22]	- Chlorination-based models[5, 14, 23] predict well, specifically, substitutions at positions 1 or 9 are highly influential.[22]	- Chlorination model predicts well,[5] substitution pattern parameters are more influential than physicochemical properties.[25]
	Analysis of toxicity	- PCDD/Fs are electron acceptors in charge transfer complexes with aryl hydrocarbon receptors, based on QSAR modeling.[8, 32]<br>- For PCDDs, molecular dynamics simulation can predict interaction energy between TCDD and AhR and rank of TEF in avian species.[33]		Toxicity is associated with<br>- high electrostatic potential[7]<br>- high chance to adopt coplanar conformation[6, 7]<br>- high chemical potential (Gibbs energy of formation per Cl atom).[27]
		Low polarity or hydrophobicity potentially leads to bioaccumulation.[7, 31, 34]		
	Method assessment	Structural data obtained from B3PW91 calculations are closer to experimental values than those from B3LYP calculations.[3]	Compared to G3XMP2 and MP2, B3LYP energy calculations underbind the significance of substitutions at positions 1 and 4.[23]	
		Compared to G3XMP2, B3LYP calculations overbind vicinal Cl-atoms repulsions.[21]		

Supplementary Table S2 List of all PCDDs by substitution positions and numbering, proposed by Ballschmiter *et al*.,[36] revised by Dorofeeva *et al*.[9] (For simplicity and completeness, number 0 of each class stands for non-substituted compound.)

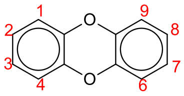

No.	Compound	No.	Compound	No.	Compound
1	1	26	2,3,7	51	1,2,3,6,7
2	2	27	1,2,3,4	52	1,2,3,6,8
3	1,2	28	1,2,3,6	53	1,2,3,6,9
4	1,3	29	1,2,3,7	54	1,2,3,7,8
5	1,4	30	1,2,3,8	55	1,2,3,7,9
6	1,6	31	1,2,3,9	56	1,2,3,8,9
7	1,7	32	1,2,4,6	57	1,2,4,6,7
8	1,8	33	1,2,4,7	58	1,2,4,6,8
9	1,9	34	1,2,4,8	59	1,2,4,6,9
10	2,3	35	1,2,4,9	60	1,2,4,7,8
11	2,7	36	1,2,6,7	61	1,2,4,7,9
12	2,8	37	1,2,6,8	62	1,2,4,8,9
13	1,2,3	38	1,2,6,9	63	1,2,3,4,6,7
14	1,2,4	39	1,2,7,8	64	1,2,3,4,6,8
15	1,2,6	40	1,2,7,9	65	1,2,3,4,6,9
16	1,2,7	41	1,2,8,9	66	1,2,3,4,7,8
17	1,2,8	42	1,3,6,8	67	1,2,3,6,7,8
18	1,2,9	43	1,3,6,9	68	1,2,3,6,7,9
19	1,3,6	44	1,3,7,8	69	1,2,3,6,8,9
20	1,3,7	45	1,3,7,9	70	1,2,3,7,8,9
21	1,3,8	46	1,4,6,9	71	1,2,4,6,7,9
22	1,3,9	47	1,4,7,8	72	1,2,4,6,8,9
23	1,4,6	48	2,3,7,8	73	1,2,3,4,6,7,8
24	1,4,7	49	1,2,3,4,6	74	1,2,3,4,6,7,9
25	1,7,8	50	1,2,3,4,7	75	1,2,3,4,6,7,8,9

Supplementary Table S3 List of all PCDFs by substitution positions and numbering, proposed by Ballschmiter *et al.*[36] (For simplicity and completeness, number 0 of each class stands for non-substituted compound.)

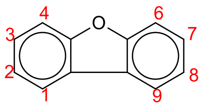

No.	Compound	No.	Compound	No.	Compound
1	1	46	2,6,7	91	1,2,3,6,7
2	2	47	3,4,6	92	1,2,3,6,8
3	3	48	3,4,7	93	1,2,3,6,9
4	4	49	1,2,3,4	94	1,2,3,7,8
5	1,2	50	1,2,3,6	95	1,2,3,7,9
6	1,3	51	1,2,3,7	96	1,2,3,8,9
7	1,4	52	1,2,3,8	97	1,2,4,6,7
8	1,6	53	1,2,3,9	98	1,2,4,6,8
9	1,7	54	1,2,4,6	99	1,2,4,6,9
10	1,8	55	1,2,4,7	100	1,2,4,7,8
11	1,9	56	1,2,4,8	101	1,2,4,7,9
12	2,3	57	1,2,4,9	102	1,2,4,8,9
13	2,4	58	1,2,6,7	103	1,2,6,7,8
14	2,6	59	1,2,6,8	104	1,2,6,7,9
15	2,7	60	1,2,6,9	105	1,3,4,6,7
16	2,8	61	1,2,7,8	106	1,3,4,6,8
17	3,4	62	1,2,7,9	107	1,3,4,6,9
18	3,6	63	1,2,8,9	108	1,3,4,7,8
19	3,7	64	1,3,4,6	109	1,3,4,7,9
20	4,6	65	1,3,4,7	110	1,3,6,7,8
21	1,2,3	66	1,3,4,8	111	1,4,6,7,8
22	1,2,4	67	1,3,4,9	112	2,3,4,6,7
23	1,2,6	68	1,3,6,7	113	2,3,4,6,8
24	1,2,7	69	1,3,6,8	114	2,3,4,7,8
25	1,2,8	70	1,3,6,9	115	1,2,3,4,6,7
26	1,2,9	71	1,3,7,8	116	1,2,3,4,6,8
27	1,3,4	72	1,3,7,9	117	1,2,3,4,6,9
28	1,3,6	73	1,4,6,7	118	1,2,3,4,7,8
29	1,3,7	74	1,4,6,8	119	1,2,3,4,7,9
30	1,3,8	75	1,4,6,9	120	1,2,3,4,8,9
31	1,3,9	76	1,4,7,8	121	1,2,3,6,7,8
32	1,4,6	77	1,6,7,8	122	1,2,3,6,7,9
33	1,4,7	78	2,3,4,6	123	1,2,3,6,8,9
34	1,4,8	79	2,3,4,7	124	1,2,3,7,8,9
35	1,4,9	80	2,3,4,8	125	1,2,4,6,7,8
36	1,6,7	81	2,3,6,7	126	1,2,4,6,7,9
37	1,6,8	82	2,3,6,8	127	1,2,4,6,8,9
38	1,7,8	83	2,3,7,8	128	1,3,4,6,7,8
39	2,3,4	84	2,4,6,7	129	1,3,4,6,7,9
40	2,3,6	85	2,4,6,8	130	2,3,4,6,7,8
41	2,3,7	86	3,4,6,7	131	1,2,3,4,6,7,8
42	2,3,8	87	1,2,3,4,6	132	1,2,3,4,6,7,9
43	2,4,6	88	1,2,3,4,7	133	1,2,3,4,6,8,9
44	2,4,7	89	1,2,3,4,8	134	1,2,3,4,7,8,9
45	2,4,8	90	1,2,3,4,9	135	1,2,3,4,6,7,8,9

Supplementary Table S4 List of all PCBs by substitution positions and numbering, first proposed by Ballschmiter *et al.*[38], later revised by IUPAC.[37] (For simplicity and completeness, number 0 of each class stands for non-substituted compound.)

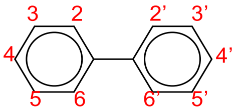

No.	Compound	No.	Compound	No.	Compound	No.	Compound	No.	Compound
1	2	43	2,2',3,5	85	2,2',3,4,4'	127	3,3',4,5,5'	169	3,3',4,4',5,5'
2	3	44	2,2',3,5'	86	2,2',3,4,5	128	2,2',3,3',4,4'	170	2,2',3,3',4,4',5
3	4	45	2,2',3,6	87	2,2',3,4,5'	129	2,2',3,3',4,5	171	2,2',3,3',4,4',6
4	2,2'	46	2,2',3,6'	88	2,2',3,4,6	130	2,2',3,3',4,5'	172	2,2',3,3',4,5,5'
5	2,3	47	2,2',4,4'	89	2,2',3,4,6'	131	2,2',3,3',4,6	173	2,2',3,3',4,5,6
6	2,3'	48	2,2',4,5	90	2,2',3,4',5	132	2,2',3,3',4,6'	174	2,2',3,3',4,5,6'
7	2,4	49	2,2',4,5'	91	2,2',3,4',6	133	2,2',3,3',5,5'	175	2,2',3,3',4,5',6
8	2,4'	50	2,2',4,6	92	2,2',3,5,5'	134	2,2',3,3',5,6	176	2,2',3,3',4,6,6'
9	2,5	51	2,2',4,6'	93	2,2',3,5,6	135	2,2',3,3',5,6'	177	2,2',3,3',4,5',6'
10	2,6	52	2,2',5,5'	94	2,2',3,5,6'	136	2,2',3,3',6,6'	178	2,2',3,3',5,5',6
11	3,3'	53	2,2',5,6'	95	2,2',3,5',6	137	2,2',3,4,4',5	179	2,2',3,3',5,6,6'
12	3,4	54	2,2',6,6'	96	2,2',3,6,6'	138	2,2',3,4,4',5'	180	2,2',3,4,4',5,5'
13	3,4'	55	2,3,3',4	97	2,2',3,4',5'	139	2,2',3,4,4',6	181	2,2',3,4,4',5,6
14	3,5	56	2,3,3',4'	98	2,2',3,4',6'	140	2,2',3,4,4',6'	182	2,2',3,4,4',5,6'
15	4,4'	57	2,3,3',5	99	2,2',4,4',5	141	2,2',3,4,5,5'	183	2,2',3,4,4',5',6
16	2,2',3	58	2,3,3',5'	100	2,2',4,4',6	142	2,2',3,4,5,6	184	2,2',3,4,4',6,6'
17	2,2',4	59	2,3,3',6	101	2,2',4,5,5'	143	2,2',3,4,5,6'	185	2,2',3,4,5,5',6
18	2,2',5	60	2,3,4,4'	102	2,2',4,5,6'	144	2,2',3,4,5',6	186	2,2',3,4,5,6,6'
19	2,2',6	61	2,3,4,5	103	2,2',4,5',6	145	2,2',3,4,6,6'	187	2,2',3,4',5,5',6
20	2,3,3'	62	2,3,4,6	104	2,2',4,6,6'	146	2,2',3,4',5,5'	188	2,2',3,4',5,6,6'
21	2,3,4	63	2,3,4',5	105	2,3,3',4,4'	147	2,2',3,4',5,6	189	2,3,3',4,4',5,5'
22	2,3,4'	64	2,3,4',6	106	2,3,3',4,5	148	2,2',3,4',5,6'	190	2,3,3',4,4',5,6
23	2,3,5	65	2,3,5,6	107	2,3,3',4,5'	149	2,2',3,4',5',6	191	2,3,3',4,4',5',6
24	2,3,6	66	2,3',4,4'	108	2,3,3',4,6	150	2,2',3,4',6,6'	192	2,3,3',4,5,5',6
25	2,3',4	67	2,3',4,5	109	2,3,3',4',5	151	2,2',3,5,5',6	193	2,3,3',4',5,5',6
26	2,3',5	68	2,3',4,5'	110	2,3,3',4',6	152	2,2',3,5,6,6'	194	2,2',3,3',4,4',5,5'
27	2,3',6	69	2,3',4,6	111	2,3,3',5,5'	153	2,2',4,4',5,5'	195	2,2',3,3',4,4',5,6
28	2,4,4'	70	2,3',4',5	112	2,3,3',5,6	154	2,2',4,4',5,6'	196	2,2',3,3',4,4',5,6'
29	2,4,5	71	2,3',4',6	113	2,3,3',5',6	155	2,2',4,4',6,6'	197	2,2',3,3',4,4',6,6'
30	2,4,6	72	2,3',5,5'	114	2,3,4,4',5	156	2,3,3',4,4',5	198	2,2',3,3',4,5,5',6
31	2,4',5	73	2,3',5',6	115	2,3,4,4',6	157	2,3,3',4,4',5'	199	2,2',3,3',4,5,5',6'
32	2,4',6	74	2,4,4',5	116	2,3,4,5,6	158	2,3,3',4,4',6	200	2,2',3,3',4,5,6,6'
33	2,3',4'	75	2,4,4',6	117	2,3,4',5,6	159	2,3,3',4,5,5'	201	2,2',3,3',4,5',6,6'
34	2,3',5'	76	2,3',4',5'	118	2,3',4,4',5	160	2,3,3',4,5,6	202	2,2',3,3',5,5',6,6'
35	3,3',4	77	3,3',4,4'	119	2,3',4,4',6	161	2,3,3',4,5',6	203	2,2',3,4,4',5,5',6
36	3,3',5	78	3,3',4,5	120	2,3',4,5,5'	162	2,3,3',4',5,5'	204	2,2',3,4,4',5,6,6'
37	3,4,4'	79	3,3',4,5'	121	2,3',4,5',6	163	2,3,3',4',5,6	205	2,3,3',4,4',5,5',6
38	3,4,5	80	3,3',5,5'	122	2,3,3',4',5'	164	2,3,3',4',5',6	206	2,2',3,3',4,4',5,5',6
39	3,4',5	81	3,4,4',5	123	2,3',4,4',5'	165	2,3,3',5,5',6	207	2,2',3,3',4,4',5,6,6'
40	2,2',3,3'	82	2,2',3,3',4	124	2,3',4',5,5'	166	2,3,4,4',5,6	208	2,2',3,3',4,5,5',6,6'
41	2,2',3,4	83	2,2',3,3',5	125	2,3',4',5',6	167	2,3',4,4',5,5'	209	2,2',3,3',4,4',5,5',6,6'
42	2,2',3,4'	84	2,2',3,3',6	126	3,3',4,4',5	168	2,3',4,4',5',6		

Supplementary Table S5 List of congener groups of PCDDs, PCDFs and PCBs by degree of substitution. The number of toxic congeners (See Table S10.) is given in red in a pair of parentheses. The number of congeners is doubled when moved from PCDDs to PCDFs for odd degree of substitution.

**Degree of chlorination**	**PCDDs**		**PCDFs**		**PCBs**	
0	1		1		1	
1	2		4		3	
2	10		16		12	
3	14		28		24	
4	22	(1)	38	(1)	42	(2)
5	14	(1)	28	(2)	46	(5)
6	10	(3)	16	(4)	42	(4)
7	2	(1)	4	(2)	24	(1)
8	1	(1)	1	(1)	12	
9	N/A		N/A		3	
10	N/A		N/A		1	
Total	76	(7)	136	(10)	210	(12)

Supplementary Table S6 Values for constant C_0 and coefficients C_1, C_2, C_3,…, parameters x_1, x_2, x_3,… and correlation coefficients for energy prediction model $E = C_0 + C_1x_1 + C_2x_2 + C_3x_3 + …$, where E is the predicted ΔG at 300K in Hartree from HF, B3LYP and electronic energy in Hartree from MP2. Pearson's correlation coefficient (R) = 1.000 for all models.

		HF		B3LYP		MP2	
PCDD	i	C_i	x_i	C_i	x_i	C_i	x_i
	0	-4280.30		-4289.65		-4284.25	
	1	458.92	total H	459.62	total H	459.12	total H
	2	3.44×10^{-3}	*o* - total	3.41×10^{-3}	*o* - total	1.89×10^{-3}	*o* - total
	3	2.45×10^{-3}	*o* - Cl-Cl	1.33×10^{-3}	*o* - Cl-Cl	9.37×10^{-4}	*o* - Cl-Cl
	4	1.33×10^{-3}	*m* - Cl-Cl	1.07×10^{-3}	*m* - Cl-Cl	5.50×10^{-4}	*p* - Cl-Cl
	5	8.48×10^{-4}	*p* - Cl-Cl			7.01×10^{-4}	*m* - Cl-Cl
	ρ	0.9998		0.9995		0.9997	
PCDF	i	C_i	x_i	C_i	x_i	C_i	x_i
	0	-4205.40		-4214.39		-4209.16	
	1	458.93	total H	459.63	total H	459.12	total H
	2	1.30×10^{-2}	CR - C	1.11×10^{-2}	CR - C	1.19×10^{-2}	CR - C
	3	6.28×10^{-3}	*o* - Cl-Cl	1.01×10^{-4}	*o* - total	3.14×10^{-3}	*o* - Cl-Cl
	4	2.39×10^{-3}	*o* - Cl-O	5.25×10^{-3}	*o* - Cl-Cl	1.15×10^{-3}	*o* - Cl-O
	5	1.23×10^{-3}	*m* - Cl-Cl	2.14×10^{-3}	*o* - Cl-O	-1.65×10^{-3}	*o* - Cl-C
	6	8.40×10^{-4}	*p* - Cl-C	9.90×10^{-4}	*m* - Cl-Cl	-8.46×10^{-4}	*m* - Cl-O
	7			-8.96×10^{-4}	*m* - Cl-O	6.51×10^{-4}	*m* - Cl-Cl
	8			1.09×10^{-3}	*p* - Cl-Cl	6.36×10^{-4}	*p* - Cl-Cl
	ρ	0.9997		0.9997		0.9997	
PCB	i	C_i	x_i	C_i	x_i	C_i	x_i
	0	-5049.56		-5059.63		-5053.47	
	1	458.94	total H	459.64	total H	459.12	total H
	2	4.36×10^{-3}	*o* - total	4.06×10^{-3}	*o* - total	3.11×10^{-3}	*o* - Cl-Cl
	3	2.10×10^{-3}	*o* - Cl-Cl	1.21×10^{-3}	*o* - Cl-Cl	4.18×10^{-4}	*m* - total
	4	1.45×10^{-3}	*m* - total	1.11×10^{-3}	*m* - total	5.05×10^{-4}	*p* - total
	5	1.06×10^{-3}	*p* - total	-6.19×10^{-4}	CR - C		
	6			8.06×10^{-4}	*p* - total		
	ρ	0.9998		0.9995		0.9977	

Supplementary Table S7 Summary of experimental data on abundance of PCDDs, PCDFs and PCBs in the literature

<table>
<tr><th colspan="2">Compound / Classification</th><th>PCDD and PCDF</th><th>PCB</th></tr>
<tr><td rowspan="2">Source</td><td>Incineration</td><td>Incineration plant emissions (Germany[36, 39, 42], Japan[43], Sweden[22])
Iron ore sinter plant (UK) and sinter plot (Swinden laboratories, Moorgate, Rotherham, UK)[54]
Pine wood combustion (USA)[40]
Automobile exhaust (Germany)[36]</td><td>Flue gas (Japan[46, 48], Sweden[25])</td></tr>
<tr><td>Commercial product</td><td>Aroclor (USA)[37]
Phenoclor (France)[37]
Clophen (Germany)[37]</td><td>Aroclor (USA)[37, 38, 41, 44, 51]
Clophen (Germany)[38]
Kaneclor (Japan)[46]</td></tr>
<tr><td rowspan="4">Acumulation</td><td rowspan="2">Environmental</td><td>Urban dust (Washington D.C., USA)[36]
Sediment (Baltic sea)[50]
Water, soil, sediment (Japan)[43]
Air, water, soil, sediment[#49]</td><td>Air, water, soil, sediment[#49]</td></tr>
<tr><td colspan="2">Residential dust (Australia)[53]</td></tr>
<tr><td rowspan="2">Biotic and food</td><td>Human tissue (New York, USA[36],[#49])
Herring (Baltic sea)[36]</td><td>Human milk[#44]
Food and animal feed[#49]</td></tr>
<tr><td colspan="2">Zucchini (Chile)[52]
Pine needles (Tokyo bay)[45]
Human milk (Guangdong, China)[55]</td></tr>
</table>

[#] Review from multiple locations

Supplementary Table S8 Rank correlation coefficients (ρ values) for association between predicted distribution (using Boltzmann distribution) of PCDDs and PCDFs based on relative stability and observed distribution in fly ash samples from municipal waste incinerators (MWI).[43] Values used for relative stability are HF, B3LYP ΔG at 600K and MP2 electronic energy. As abundance values were derived from GC/MS chromatograms, values from co-eluting peaks were discarded from calculations.

	Congener group	Number of data points	Sample[a]	HF	B3LYP	MP2
**PCDDs**	Tetrachlorodibenzodioxin ($C_{12}H_4O_2Cl_4$)	10	4	0.7048	0.6585	0.6810
			5	0.4956	0.4499	0.5077
			6	0.9428	0.8550	0.8882
			7	0.4576	0.3547	0.3969
	Pentachlorodibenzodioxin ($C_{12}H_3O_2Cl_5$)	10	4	0.6414	0.5988	0.6563
			5	0.5833	0.6341	0.6667
			6	N/A	N/A	N/A
			7	0.7871	0.6031	0.7550
	Hexachlorodibenzodioxin ($C_{12}H_2O_2Cl_6$)	5	4	0.4104	0.5526	0.6316
			5	0.5000	0.6156	0.6669
			6	0.8000	0.8721	0.8721
			7	0.5000	0.6156	0.6669
	Heptachlorodibenzodioxin ($C_{12}HO_2Cl_7$)	2	4	-1.0000	-1.0000	-1.0000
			5	-1.0000	-1.0000	-1.0000
			6	-1.0000	-1.0000	-1.0000
			7	-1.0000	-1.0000	-1.0000
	Median for each method (*n*=15)		All	0.5000	0.5988	0.6563
**PCDFs**	Tetrachlorodibenzofuran ($C_{12}H_4OCl_4$)	6	4	-0.3714	-0.3714	-0.3143
			5	-0.6571	-0.6571	-0.6000
			6	0.3714	0.3714	0.4286
			7	0.0857	0.0857	0.1429
	Pentachlorodibenzofuran ($C_{12}H_3OCl_5$)	8	4	0.0120	0.0120	0.2515
			5	-0.0238	-0.0238	0.1905
			6	0.7306	0.7306	0.7545
			7	0.0238	0.0238	0.2619
	Hexachlorodibenzofuran ($C_{12}H_2OCl_6$)	10	4	0.7697	0.7091	0.8061
			5	0.7295	0.6383	0.7599
			6	0.8632	0.7781	0.8268
			7	0.7939	0.6727	0.7697
	Heptachlorodibenzofuran ($C_{12}HOCl_7$)	4	4	0.8000	0.8000	0.8000
			5	0.8000	0.8000	0.8000
			6	1.0000	0.4000	1.0000
			7	1.0000	0.4000	1.0000
	Median for each method (*n*=16)		All	0.7300	0.4000	0.7572

[a]See sample descriptions in the reference

For PCBs, data were compared to observed distribution from 6 combustion experiments.[25] ρ values are provided in an excel file (PCB/PCB_isomerdist_correlation.xlsx).

Supplementary Table S9 Values for constant C_0, coefficients C_1, C_2 and correlation coefficients for the PCDF coplanarity prediction model: $A = C_0 + (x_1 x_9)C_1 + (x_2 x_8)C_2$, where A is the predicted angle value (see manuscript for further description) and x_i is 1 if compound is chlorinated at the position i and 0 if otherwise. Predictors are all statistically significant (p<.05). Models based on HF calculations are not given as according to HF results, only two PCDFs are nonplanar.

	**Angle group**	**Coefficients**			R	ρ
		C_0	C_1	C_2		
B3LYP	1	-0.0649	0.3029	0.2595	0.6446	0.4481
	2	-0.3591	1.6806	1.4366	0.6457	0.4481
MP2	1	-0.0440	0.1808	0.1760	0.6100	0.5385
	2	-0.2317	0.9508	0.9267	0.6117	0.5385

Supplementary Table S10 List of toxic equivalency factor (TEF) values of 7 PCDDs, 10 PCDFs and 12 PCBs adapted from Van den Berg *et al*.[57]

		Compound No.	(Substitution positions)	WHO 1998 TEF	WHO 2005 TEF
**PCDDs**	(Table S2)	48	(2,3,7,8)	1	1
		54	(1,2,3,7,8)	1	1
		66	(1,2,3,4,7,8)	0.1	0.1
		67	(1,2,3,6,7,8)	0.1	0.1
		70	(1,2,3,7,8,9)	0.1	0.1
		73	(1,2,3,4,6,7,8)	0.01	0.01
		75	(1,2,3,4,6,7,8,9)	0.0001	0.0003
**PCDFs**	(Table S3)	83	(2,3,7,8)	0.1	0.1
		94	(1,2,3,7,8)	0.05	0.03
		114	(2,3,4,7,8)	0.5	0.3
		118	(1,2,3,4,7,8)	0.1	0.1
		121	(1,2,3,6,7,8)	0.1	0.1
		124	(1,2,3,7,8,9)	0.1	0.1
		130	(2,3,4,6,7,8)	0.1	0.1
		131	(1,2,3,4,6,7,8)	0.01	0.01
		134	(1,2,3,4,7,8,9)	0.01	0.01
		135	(1,2,3,4,6,7,8,9)	0.0001	0.0003
**PCBs**	(Table S4)	77	(3,3’,4,4’)	0.0001	0.0001
		81	(3,4,4’,5)	0.0001	0.0003
		105	(2,3,3’,4,4’)	0.0001	0.00003
		114	(2,3,4,4’,5)	0.0005	0.00003
		118	(2,3’,4,4’,5)	0.0001	0.00003
		123	(2,3’,4,4’,5’)	0.0001	0.00003
		126	(3,3’,4,4’,5)	0.1	0.1
		156	(2,3,3’,4,4’,5)	0.0005	0.00003
		157	(2,3,3’,4,4’,5’)	0.0005	0.00003
		167	(2,3’,4,4’,5,5’)	0.00001	0.00003
		169	(3,3’,4,4’,5,5’)	0.01	0.03
		189	(2,3,3’,4,4’,5,5’)	0.0001	0.00003
